# Controversies in Vitamin D: A Statement From the Third International Conference

**DOI:** 10.1002/jbm4.10417

**Published:** 2020-11-10

**Authors:** Andrea Giustina, Roger Bouillon, Neil Binkley, Christopher Sempos, Robert A Adler, Jens Bollerslev, Bess Dawson‐Hughes, Peter R Ebeling, David Feldman, Annemieke Heijboer, Glenville Jones, Christopher S Kovacs, Marise Lazaretti‐Castro, Paul Lips, Claudio Marcocci, Salvatore Minisola, Nicola Napoli, Rene Rizzoli, Robert Scragg, John H White, Anna Maria Formenti, John P Bilezikian

**Affiliations:** ^1^ Institute of Endocrine and Metabolic Sciences, San Raffaele, Vita‐Salute University and IRCCS Hospital Milan Italy; ^2^ Laboratory of Clinical and Experimental Endocrinology, Department of Chronic Diseases Metabolism and Ageing KU Leuven Leuven Belgium; ^3^ Osteoporosis Clinical Research Program on Aging, University of Wisconsin Madison WI USA; ^4^ Vitamin D Standardization Program LLC Havre de Grace MD USA; ^5^ McGuire Veterans Affairs Medical Center and Virginia Commonwealth University School of Medicine Richmond VA USA; ^6^ Section of Specialized Endocrinology, Department of Endocrinology, Oslo University Hospital, Rikshospitalet, Oslo, Norway, and Faculty of Medicine University of Oslo Oslo Norway; ^7^ Jean Mayer USDA Nutrition Research Center on Aging Tufts University Boston MA USA; ^8^ Department of Medicine, School of Clinical Sciences Monash University Calyton Victoria Australia; ^9^ Department of Medicine Stanford University School of Medicine Stanford CA USA; ^10^ Endocrine Laboratory, Department of Clinical Chemistry Amsterdam UMC, Vrije Universiteit Amsterdam and University of Amsterdam, Amsterdam Gastroenterology & Metabolism Amsterdam The Netherlands; ^11^ Department of Biomedical and Molecular Sciences Queen's University Kingston Ontario Canada; ^12^ Faculty of Medicine Memorial University of Newfoundland St. John's Newfoundland and Labrador Canada; ^13^ Division of Endocrinology Escola Paulista de Medicina–Universidade Federal de Sao Paulo (EPM‐UNIFESP) São Paulo Brazil; ^14^ Department of Internal Medicine, Endocrine Section Amsterdam University Medical Center Amsterdam The Netherlands; ^15^ Department of Clinical and Experimental Medicine University of Pisa Pisa Italy; ^16^ Department of Internal Medicine and Medical Disciplines University of Rome “Sapienza” Rome Italy; ^17^ Unit of Endocrinology and Diabetes Campus Bio‐Medico, University of Rome Rome Italy; ^18^ Division of Bone and Mineral Diseases Washington University in St. Louis St. Louis MO USA; ^19^ Service of Bone Diseases Geneva University Hospitals and Faculty of Medicine Geneva Switzerland; ^20^ School of Population Health University of Auckland Auckland New Zealand; ^21^ Department of Physiology McGill University Montreal Quebec Canada; ^22^ Department of Medicine, Endocrinology Division, College of Physicians and Surgeons Columbia University New York NY USA

**Keywords:** VITAMIN D, VITAMIN D DEFICIENCY, NUTRITION, ENDOCRINE PATHWAYS, OSTEOMALACIA, RICKETS, METABOLIC BONE DISEASES

## Abstract

The Third International Conference on Controversies in Vitamin D was held in Gubbio, Italy, September 10–13, 2019. The conference was held as a follow‐up to previous meetings held in 2017 and 2018 to address topics of controversy in vitamin D research. The specific topics were selected by the steering committee of the conference and based upon areas that remain controversial from the preceding conferences. Other topics were selected anew that reflect specific topics that have surfaced since the last international conference. Consensus was achieved after formal presentations and open discussions among experts. As will be detailed in this article, consensus was achieved with regard to the following: the importance and prevalence of nutritional rickets, amounts of vitamin D that are typically generated by sun exposure, worldwide prevalence of vitamin D deficiency, the importance of circulating concentrations of 25OHD as the best index of vitamin D stores, definitions and thresholds of vitamin D deficiency, and efficacy of vitamin D analogues in the treatment of psoriasis. Areas of uncertainly and controversy include the following: daily doses of vitamin D needed to maintain a normal level of 25OHD in the general population, recommendations for supplementation in patients with metabolic bone diseases, cutaneous production of vitamin D by UVB exposure, hepatic regulation of 25OHD metabolites, definition of vitamin D excess, vitamin D deficiency in acute illness, vitamin D requirements during reproduction, potential for a broad spectrum of cellular and organ activities under the influence of the vitamin D receptor, and potential links between vitamin D and major human diseases. With specific regard to the latter area, the proceedings of the conference led to recommendations for areas in need of further investigation through appropriately designed intervention trials. © 2020 The Authors. *JBMR Plus* published by Wiley Periodicals LLC. on behalf of American Society for Bone and Mineral Research.

## Introduction

Following meetings held in 2017^(^ ^1^ ^)^ and 2018,^(^ ^2^ ^)^ the Third International Conference on Controversies in Vitamin D was held in Gubbio, Italy, September 10–13, 2019. The aim of the conference was to convene leading worldwide experts in vitamin D research to address ongoing controversies and current topics of debate in vitamin D research. Following formal presentations on specific topics, discussions among experts were used to help resolve lingering issues and to clarify areas of uncertainty. Several core issues from the previous conference in 2018 were revisited, such as assays to determine serum 25OHD concentration, which remains a critical and controversial issue for defining vitamin D status. Definitions of vitamin D nutritional status were also revisited. New areas were discussed, including the epidemiology of vitamin D in developing countries and 25OHD threshold values and how they should be defined in the context of health and disease in different stages of human development. Therapeutic roles of vitamin D and findings from recent randomized clinical trials were also discussed for cancer, cardiovascular disease, and diabetes mellitus (DM). It was evident that results from recent trials are inconclusive because of questionable design, the treatment regimen adopted, or the baseline vitamin D status of the study subjects. Here we also identify issues concerning vitamin D in both skeletal and nonskeletal diseases where consensus is becoming established or is still lacking.

### Topics Considered for Consensus

### Nutritional rickets

Nutritional rickets, caused by a simple vitamin D or calcium deficiency or both, still affects a significant number of infants and children worldwide.^(^ ^3^ ^)^ Vitamin D‐deficiency rickets is cured by vitamin D administration.^(^ ^3^ ^)^ There is consensus that infants and most children require approximately 400 IU (or 600 IU for older children) of vitamin D per day to prevent rickets because direct exposure to sunlight is often avoided and not recommended for the very young.^(^ ^4^ ^)^ However, such a supplementation policy is either not or not fully implemented in many countries.

Although countries in Asia and the Middle East are most often affected by nutritional vitamin D deficiency, African and some Asian countries also encounter rickets caused by calcium deficiency.^(^ ^3^ ^)^ For newborns 0 to 6 and infants 6 to 12 months of age, adequate calcium intake is 200 and 260 mg/day, respectively, whereas for children over 12 months of age, a dietary calcium intake of <300 mg/day increases the risk of rickets independent of serum 25OHD levels.^(^ ^5^ ^)^ For children over 12 months of age, classification of dietary calcium intake can be defined as: sufficiency = >500 mg/day; insufficiency = 300 to 500 mg/day, and deficiency = <300 mg/day.^(^ ^6^ ^)^

**Fig 1:**
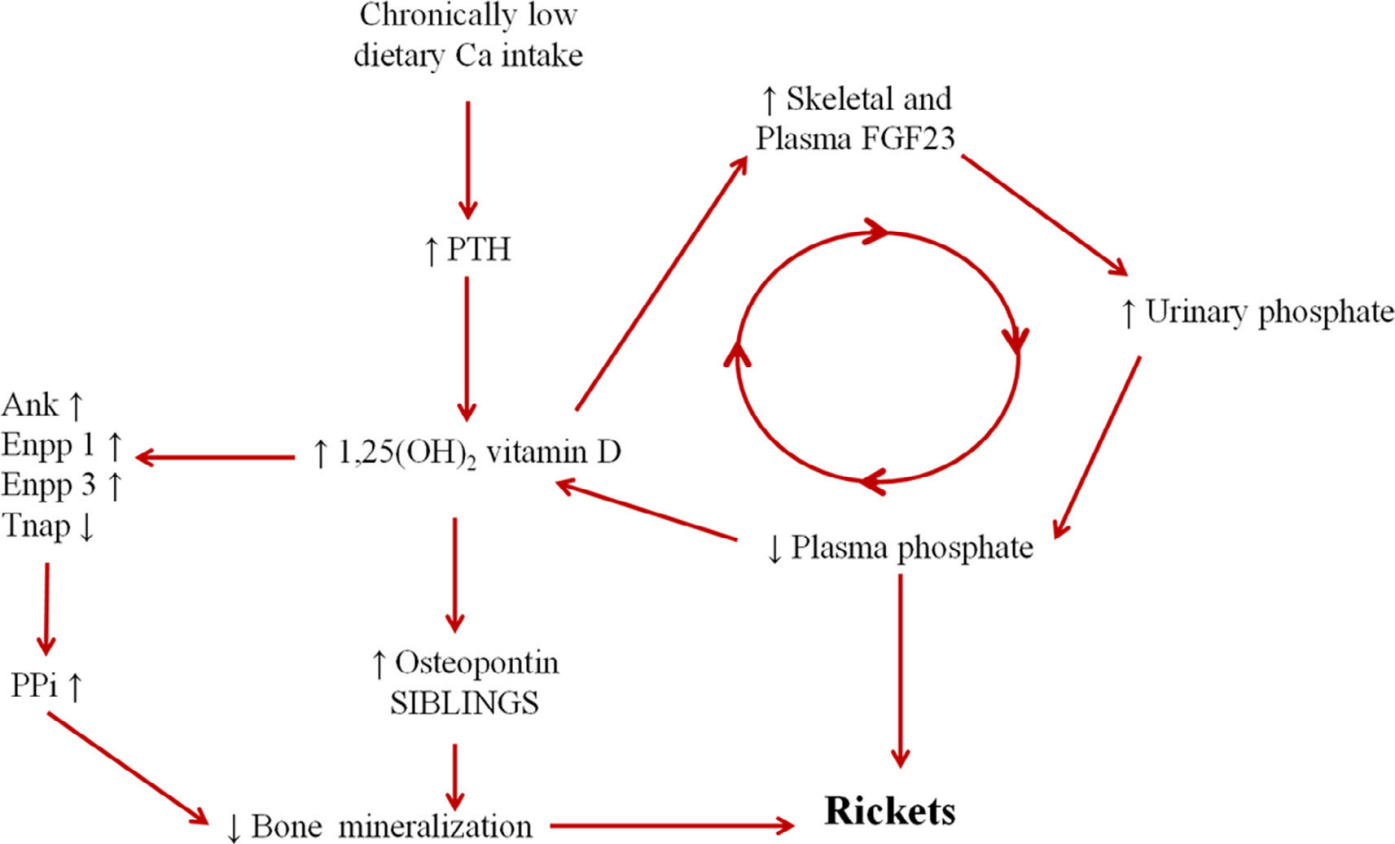
Mechanisms involved in the pathogenesis of rickets caused by a chronic low calcium intake. ANK = ankylosis protein; Ca = calcium; ENPP = ectonucleotide pyrophosphatase/phosphodiesterase; FGF‐23 = fibroblast growth factor 23; PPi = inorganic pyrophosphate; PTH = parathyroid hormone; SIBLINGS = small integrin‐binding ligand, N‐linked glycoproteins; Tnap = tissue nonspecific alkaline phosphatase.

The pathogenesis of calcium deficiency rickets is probably more complex than previously thought. However, we do know that reduced calcium intake increases PTH secretion, which in turn increases FGF‐23. Increases in both PTH and in FGF‐23 lead to an increase in urinary phosphate excretion. This pathophysiological sequence leads to reduced serum phosphate, which, along with PTH, increases the 1,25‐dihydroxyvitamin D [1,25(OH)D] level. Elevated 1,25(OH)D upregulates a number of genes causing an increase in pyrophosphate, a known inhibitor of bone mineralization, along with osteopontin and small integrin‐binding ligand N‐linked glycoproteins (SIBLINGS; Fig. 1).^(^ ^7^, ^8^, ^9^, ^10^ ^)^ These abnormalities, along with low calcium and low phosphate levels, are primarily responsible for the osteomalacia characteristic of calcium deficiency. Although this pathophysiological sequence has been demonstrated in animals, it is likely that humans are affected in the same way.

Currently, there is still a high incidence of rickets, mainly based on clinical signs, in different countries around the world (Table 1).^(^ ^11^ ^)^ Based on the widespread global prevalence of rickets, a task force should be established to deal with this problem. Such a task force comprised of representatives from societies such as the International Society of Endocrinology, the International Federation of Musculoskeletal Research Societies, the Pediatric Endocrine Society, and the European Society for Paediatric Endocrinology, as well as representatives from the vitamin D conference should prepare and present a plan to the WHO to eradicate rickets before 2030.^(^ ^3^ ^)^

**Table 1:** Prevalence of Rickets Worldwide

Country	Year	Rate (%)	Method
Mongolia	1998	70	Rickets signs
Tibet	1994	66	Rickets signs
Ethiopia	1997	42	X‐rays
Yemen	1987	27	–
Turkey	1994	10	–
Nigeria	1998	9	Rickets signs
Iran	1975	15	X‐rays
China	1977–83	47	Rickets signs
		3.7	X‐rays/biochem
The Gambia (West Kiang)	2007	3.3	Rickets signs
		0.6	Physician exam
Bangladesh (Chittagong)	2008	2.2	Rickets signs
		1.0	X‐rays

### Vitamin D is produced by UVB light from the sun

UVB light (wavelength of approximately 280 to 310 nm) opens the B ring of 7‐dehydrocholesterol, the last step in the de novo synthesis of cholesterol, and generates previtamin D, which undergoes thermally induced isomerization into vitamin D_3_ before being transferred into the circulation by binding to the serum vitamin D binding protein (DBP).

**Fig 2:**
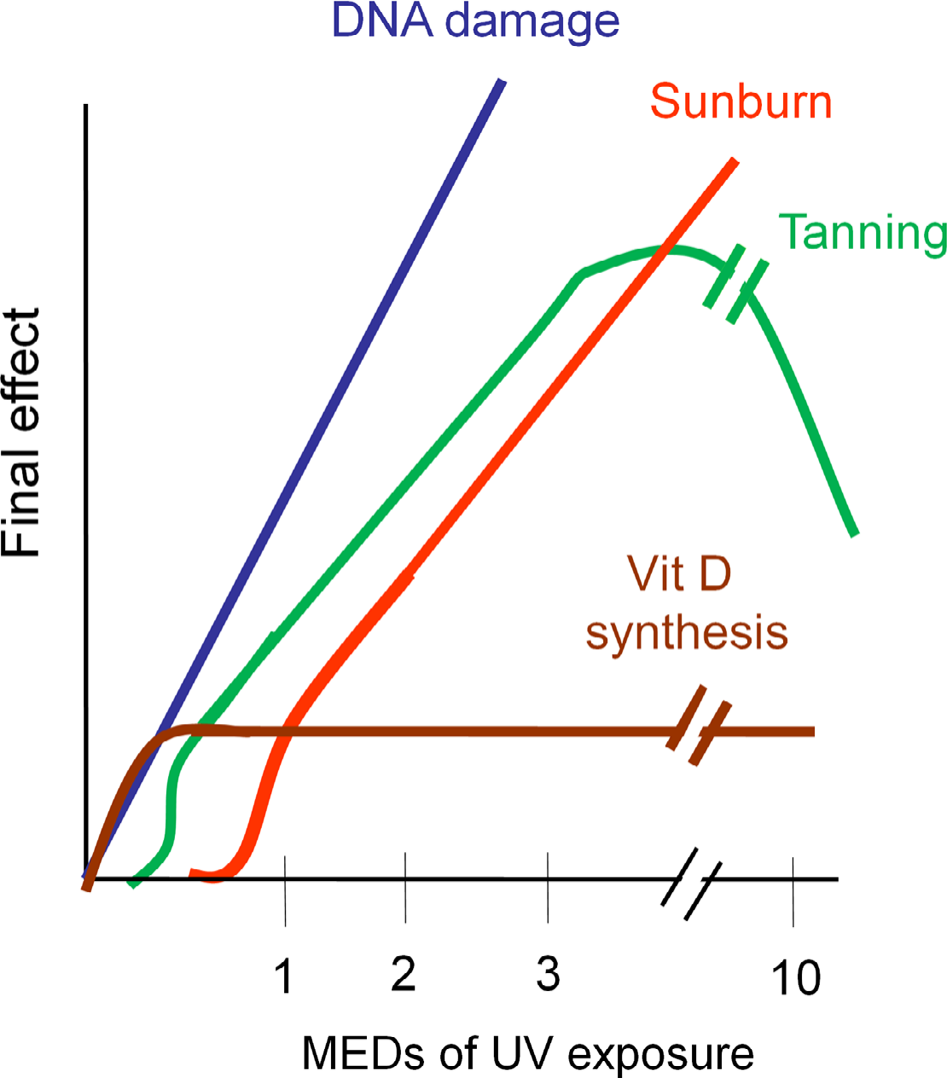
Relationship between minimal erythema dose (MED) of UV exposure and level of DNA damage, suntan/tanning, and vitamin D synthesis.

Short periods of exposure to sunlight are beneficial for vitamin D production, whereas prolonged UVB exposure leads to sunburn and DNA damage.^(^ ^12^ ^)^ Larger doses result in more intense peak reactions in a roughly linear fashion, with the actual slope of the lines defined by individual variability, which in turn is probably accounted for, at least in part, by genetic determinants. As UV doses increase, simple tanning is replaced by more advanced degrees of sunburn. In contrast, vitamin D formation is instantaneous and increases linearly in a time‐dependent fashion from very small to very large UV exposures. The dose response for dermal photosynthesis of vitamin D increases linearly at small UV doses, but differs strikingly from the other dose–response curves in reaching a plateau well below the threshold dose for erythema; Fig. 2). ^(^ ^13^, ^14^ ^)^ Thus, short UVB exposure times increase vitamin D photosynthesis. However, many other variables can influence vitamin D dermal photosynthesis such as age, skin color, sunscreen use, latitude, time of day, and season. As a result, there is no consensus on what constitutes safe and effective exposure to sunlight for the general population.^(^ ^6^ ^)^ Moreover, given the above‐noted individual differences, attempting blanket guidance seems ill‐advised.

### Vitamin D deficiency is prevalent

Although cutaneous vitamin D_3_ synthesis occurs rapidly in the presence of adequate solar UVB because of human behavior—indoor work, sun avoidance, etc.—vitamin D deficiency is widespread.^(^ ^15^ ^)^ Using a definition of <20 ng/mL (<50 nmol/L),^(^ ^16^ ^)^ as many as one third of the world's population is deficient, with a percentage as high as 40% in Europe (Table 2). Severe vitamin D deficiency, defined as <30 nmol/L (or <12 ng/mL), is seen in approximately 7% of the population worldwide, with considerable variation observed between different countries and populations. Nevertheless, severe vitamin D deficiency occurs in high‐risk populations worldwide.^(^ ^17^ ^)^ High‐risk groups for vitamin D deficiency include those who lack effective exposure to sunlight. This could be because of a variety of climatologic, cultural, or religious reasons, as well to skin pigmentation. Vitamin D deficiency was long considered rare in Africa, but a systematic analysis of African countries revealed that severe vitamin D deficiency is present in 18% of all African subjects, with clusters having a high prevalence of deficiency widely dispersed based upon cultural/behavioral practices.^(^ ^18^, ^19^, ^20^, ^21^, ^22^, ^23^, ^24^, ^25^, ^26^ ^)^

**Table 2:** Vitamin D Deficiency Around the World

Serum 25OHD	<25/30 nmol/L (12 ng/mL) (%)	<50 nmol/L (29 ng/mL) (%)
World overview^a^	6.7	37
US: NHANES 2010 data^b^ (>12 years)	6.7	26
EU countries (adults)^c^	13	40
Middle East/N Aftric, Iran + Jordan^d^	_~_50	90
African countries^e^	<0.1	7
China^f^	_~_37	_~_72
Mongolia^d^	_~_50	

^a^ Hilger et al, 2014^17^

^b^ Schleicher et al 2016^22^

^c^ Cashman et al 2017^23^

^d^ Arabi et al 2010^24^

^e^ Durazo‐Arvizu et al 2014^25^

^f^ Zhang et al 2013^26^

### 25OHD is the “best” marker of vitamin D status

The circulating 25OHD concentration is widely accepted as the best marker of an individual's vitamin D status, and has been used by numerous agencies in the establishment of vitamin D dietary requirements and for population surveillance of vitamin D deficiency or inadequacy.^(^ ^27^ ^)^ However, circulating 25OHD has, at least historically, been felt to have little physiologic regulation, thus other measures could potentially be better indicators of vitamin D status. Notably, there is ongoing debate with regard to whether free 25OHD (unbound to carrier proteins) or the ratio of 24,25‐dihydroxyvitamin D [24,25(OH)_2_D]:25OHD is a superior marker than total 25OHD.^(^ ^28^ ^)^

The ratio of 25(OH)D_3_:24,25(OH)_2_D_3_ has been developed as a diagnostic tool for idiopathic infantile hypercalcemia caused by mutations of CYP24A1. However, the ratio is also elevated in patients with vitamin D deficiency, who undergo dialysis for chronic kidney disease caused by downregulation of the CYP24A1 enzyme.^(^ ^29^, ^30^ ^)^ It is also possible in certain circumstances that the ratio of 1,25(OH)_2_D:25OHD could be a useful marker for CYP27B1 activity.^(^ ^31^ ^)^

Importantly, vitamin D research data are plagued by variation in the quality of serum total 25OHD assay methods—which has compromised, and continues to compromise—the ability to distinguish among the different guidelines currently in use.^(^ ^32^ ^)^ Similarly, uncertainty about the quality of free 25OHD measurement hinders its evaluation compared with serum total 25OHD. For 25OHD and 24,25(OH)D_2_, reference methods are available that are used to improve the standardization of these analytes. Standardization is encouraged by the Vitamin D Standardization Program (VDSP) and by the Vitamin D External Quality Assurance Scheme (DEQAS). DEQAS, backed‐up by CDC‐standardized target values, has monitored the performance of 700 to 1000 laboratories assaying 25OHD quarterly for 30 years. Over the decades, it has documented problematic assays and kit manufacturers.^(^ ^33^, ^34^ ^)^ DEQAS also promotes an accurate assay of 24,25(OH)_2_D_3_ and 1,25(OH)_2_D by circulating serum samples.

Currently, the VDSP is coordinating an effort to harmonize direct free 25OHD measurement by the development of “trueness” controls (Personal Communication, Professor Chris T Sempos). Finally, the NIH Office of Dietary Supplements, as part of the VDSP, is sponsoring the development of a reference method for 1,25(OH)_2_D, which will help to standardize its measurement in vitamin D research and bring clarity to its role.^(^ ^26^ ^)^ Such standardization efforts are essential to advance clarification of what truly constitutes vitamin D deficiency.

However, standardization is not the only analytical challenge in the measurement of vitamin D metabolites. Patient‐ or matrix‐dependent deviations are a well‐known confounder in many 25OHD immunoassays leading to inaccurate results, for example, in pregnant women or hemodialysis patients. In addition, differences in the affinity for, or release from DBP for 25(OH)D_3_ and 25(OH)D_2_ within immunoassays lead to important problems in the determination of the serum 25OHD concentration in subjects taking D2 supplements.^(^ ^35^ ^)^ These problems cannot be solved by standardization initiatives, but are inherent in the specific immunoassays; it is therefore essential that these immunoassays should be improved as well. This is particularly important in regions where ergocalciferol is commonly used and for vegans who may choose to avoid cholecalciferol.

### Definition/thresholds of vitamin D deficiency

There is an ongoing debate regarding the definition of vitamin D deficiency as noted by different recommendations from various expert groups.^(^ ^4^ ^)^ However, there is consensus on two points: 25OHD levels below 12 ng/mL (30 nmol/L) are clearly deficient at all ages and levels above 30 ng/mL (75 nmol/L) are clearly sufficient. In contrast, there is disagreement on how to regard levels between 12 and 30 ng/mL (30 and 75 nmol/L). Some guidelines recommend a threshold value of 20 ng/mL (50 nmol/L),^(^ ^36^ ^)^ whereas others aim for ≥30 ng/mL (≥ 75 nmol/L).^(^ ^37^ ^)^ This discussion is based in large part on the lack of 25OHD assay standardization.^(^ ^32^ ^)^

These cut points have key implications for randomized clinical trials (RCTs). There are few clinical trials that enrolled clearly vitamin D‐deficient subjects; one example is the work of Chapuy and colleagues.^(^ ^38^, ^39^ ^)^ The importance of studying the effect(s) of vitamin D supplementation only in deficient subjects cannot be overemphasized because vitamin D is a threshold nutrient,^(^ ^40^ ^)^ which means that a physiological endpoint, such as calcium absorption, is enhanced in dose–response fashion up to the threshold value above which higher levels do not lead to a greater effect. If a clinical trial enrolls subjects whose 25OHD levels are above the threshold, randomizing subjects to receive additional vitamin D greatly reduces the likelihood of showing a benefit of supplementation. Recent well‐publicized RCTs published in major peer‐reviewed journals illustrate this confounding point well.^(^ ^41^, ^42^, ^43^, ^44^ ^)^ One would not expect to see an effect of a threshold nutrient if both the control and the supplemented groups started at baseline with sufficient levels of 25OHD.^(^ ^45^ ^)^

### Vitamin D analogues are the preferred local therapy for psoriasis

The benefits of vitamin D analogues for the treatment of psoriasis are well‐established.^(^ ^46^, ^47^, ^48^ ^)^ A topical vitamin D analogue is a first‐line choice in the management of psoriasis, either alone or in combination with topical corticosteroids.^(^ ^49^, ^50^, ^51^, ^52^, ^53^, ^54^ ^)^ Unlike corticosteroids, which can be associated with tachyphylaxis, topically administered vitamin D analogue treatment is effective long‐term without side‐effects in patients of all ages.^(^ ^55^, ^56^, ^57^, ^58^, ^59^ ^)^

## Topics for Which Consensus Is Not Established

### What daily doses of vitamin D are recommended to maintain a normal level of 25OHD in the general population?

The Institute of Medicine recommends 400–600–800 IU/day vitamin D supplementation if there is no exposure to sunlight for infants, children/adults, and elderly, respectively.^(^ ^36^ ^)^ These recommendations are endorsed by guidelines formulated by Nordic and DACH (German‐speaking) countries, Australia and New Zealand, the European Food Safety Agency, the European Calcified Tissue Society, and the International Osteoporosis Foundation.^(^ ^60^, ^61^, ^62^, ^63^, ^64^, ^65^ ^)^ The Endocrine Society recommends 600 IU/day up to 2000 IU/day for so‐called risk groups.^(^ ^37^ ^)^ UK guidelines (the Scientific Advisory Committee on Nutrition) recommend 400 IU/day for any age.^(^ ^66^ ^)^ A few other organizations suggest much higher doses (4000 to 10,000 IU/day).^(^ ^67^ ^)^ These recommendations are for individuals who do not have osteoporosis or other metabolic bone disease. Unfortunately, this point has not been appreciated by many organizations or practitioners. Errors can occur in two ways. First, subjects who are overly concerned about their skeletal health could conceivably take too much if recommendations by some bodies of up to 10,000 IU per day are followed. It is estimated, for example, that 3% of adults in the United States take a vitamin D supplement of >4000 IU/day.^(^ ^68^ ^)^ Such amounts could potentially be deleterious as such doses may decrease rather than increase BMD or bone strength.^(^ ^69^ ^)^ On the other hand, use of relatively low doses could be deleterious for those in whom requirements are higher (eg, malabsorption or obesity). It is clearly essential to define and reach consensus regarding what constitutes deficiency to allow resolution of existing differences in daily supplementation dose recommendations.

### What are the recommendations for supplementation in patients with metabolic bone diseases?

In patients who have osteoporosis or other metabolic bone diseases, the discussion about vitamin D is different from that for the general population.^(^ ^70^ ^)^ Clearly, greater emphasis is placed upon first ensuring that the 25OHD level is sufficiently above the threshold, whichever one is being followed, either 20 or 30 ng/mL (50 or 75 nmol/L). Furthermore, there is evidence that the response to antiosteoporosis drugs may be enhanced when vitamin D and calcium sufficiency are ensured.^(^ ^71^, ^72^ ^)^

This consensus has recently been questioned by a meta‐analysis conducted by Bolland and colleagues.^(^ ^73^ ^)^ In their review, they stated: “Our findings suggest that vitamin D supplementation does not prevent fractures or falls or have clinically meaningful effects on bone mineral density.” They concluded their discussion with the following statement: “There is little justification to use vitamin D supplements to maintain or improve musculoskeletal health” and “This conclusion should be reflected in clinical guidelines.”^(^ ^73^ ^)^ First, other experts, who have taken issue with their statements, have questioned such conclusions. Lips, Bilezikian, and Bouillon^(^ ^74^ ^)^ note that this meta‐analysis excluded all studies that compared calcium plus vitamin D versus double placebo. Boonen and colleagues showed many years ago that it is necessary to administer both calcium and vitamin D in sufficient amounts to observe a reduction in fractures.^(^ ^75^ ^)^ Weaver and colleagues, representing the National Osteoporosis Foundation, came to the same conclusion,^(^ ^76^ ^)^ as did Yao and colleagues in a recent meta‐analysis.^(^ ^77^ ^)^ Second, over 60% of the studies were short‐term, <1 year. It is unreasonable to expect a beneficial effect of antiosteoporotic nutrients on fracture risk over such a short period. Third, vitamin D‐deficient individuals (25OHD <12 ng/mL or 30 nmol/L) represented a miniscule percentage of the entire population studied: <2.1%. Fourth, the trial that constituted individuals at highest fracture risk (18%) was hampered by poor compliance (~50%).^(^ ^78^ ^)^ Another flaw in this meta‐analysis was inclusion of studies that utilized high intermittent boluses of vitamin D, which might increase fracture risk.^(^ ^79^ ^)^ Moreover, the two main authors of this meta‐analysis have independently published separate meta‐analyses in which they conclude that combined vitamin D and calcium supplements can reduce the risks of hip and nonvertebral fractures in the elderly.^(^ ^73^, ^80^ ^)^ This earlier review was not mentioned or discussed in their latest meta‐analysis. Other experts have reached similar conclusions.^(^ ^74^, ^81^ ^)^ Nevertheless, the debate is alive with contrary views still being expressed as recently as the 2019 meeting of the ASBMR.^(^ ^82^ ^)^

### Cutaneous production of vitamin D by UVB exposure

Studies led by Holick and colleagues have repeatedly stated that a full day of sun exposure can produce 10,000 to 25,000 IU of vitamin D.^(^ ^83^, ^84^, ^85^ ^)^ To this point, in other studies it has been shown that the indigenous, very dark‐skinned Masai people are said to make 10,000 or 20,000 IU per day. More recent studies have raised questions about the magnitude of the sun's effect on dermal vitamin D production. Young Danish women exposed to intensive sun in the Canary Islands showed an increase in 25OHD that was equivalent to only 600 to 1000 IU/per day. A similar increase in serum 25OHD was induced by comparing total‐body UVB exposure three times per week with an oral daily intake of only 800 IU of vitamin D.^(^ ^86^, ^87^ ^)^ Another study from the Canary Islands of young Danish women, exposed to 1 week of daily sunlight, showed that serum levels of 25OHD increased by only 20 nmol/L (8 ng/mL), equivalent to about 800 IU of oral vitamin D per day.^(^ ^88^ ^)^ In yet another study from the Canary Islands, young Polish volunteers with near total body sun exposure achieved a change in 25OHD of 28 nmol/L or approximately 12 ng/mL equivalent to approximately 600 to 1200 IU (~15 to 30 μg) of oral vitamin D per day.^(^ ^89^ ^)^ Finally, exposure of 1000 cm^2^ on the back three times per week at half the minimal erythematous dose in nursing home residents increased median serum 25OHD in 3 months from 7.2 to 24 ng/mL (18 nmol/L to 60 nmol/L), equivalent to a supplement of 400 IU/day.^(^ ^90^ ^)^ It is at this time unclear what full daily exposure to sun produces. Is it about 1000 IU or closer to 10,000 IU? An answer to that question may be helpful in the interpretation of the daily requirements of vitamin D in subjects with little exposure to sunlight.

### Hepatic regulation of 25OHD metabolism

Biochemical dogma states that vitamin D is hydroxylated in the liver to 25OHD by the constitutively active hydroxylase, CYP2R1. Although this enzyme is clearly the major converting enzyme, it is not the only way in which hydroxylation occurs.^(^ ^91^ ^)^ Recent data have also called into question the constitutive nature of this reaction by evidence suggesting this enzyme is subject to several different control mechanisms. For example, in the fasting state, a significant reduction in the expression of CYP2R1 can be observed.^(^ ^92^ ^)^ In a murine model of DM, a 50% reduction in mRNA and protein expression of CYP2R1 is demonstrable.^(^ ^92^ ^)^ This study identified novel molecular mechanisms (involving PPAR γ coactivator a1 and estrogen‐related receptor) for vitamin D deficiency in DM and showed a novel negative feedback mechanism that controls cross‐talk between energy homeostasis and the vitamin D pathway. Activation of the glucocorticoid receptor (by dexamethasone or other corticosteroids) also supresses the activity of CYP2R1. Thus, rather than viewing the liver as a constitutive factory for the quantitative conversion of vitamin D to 25OHD via an unregulated CYP2R1 enzyme, metabolic and hormonal mechanisms are operative. More research is clearly needed to understand better how the production of 25OHD is regulated in the liver.

### Definition of vitamin D excess

The classical concept of vitamin D toxicity was thought to be that level above which hypercalcemia was likely to occur. Serum 25OHD values in excess of 100 or 150 ng/mL (250 or 375 nmol/L) may lead to hypercalcemia and, thus, these cut points became frames of reference for a number of authoritative bodies, such as the Institute of Medicine, the Endocrine Society, and reference laboratories.^(^ ^36^, ^37^, ^93^ ^)^

Although it would seem reasonable to identify hypercalcemia as a threshold of toxicity, other indices of toxicity, such as hypercalciuria could occur at much lower levels.^(^ ^94^ ^)^ In the study by Gallagher and colleagues, hypercalciuria occurred in 30% of vitamin D‐deficient individuals administered only 800 to 2000 IU per day for 1 year, whereas hypercalcemia occurred in 9%.

Further human studies conducted by Gallagher and colleagues and reanalyzed by Kaufmann and colleagues showed that doses up to 4000 IU/day for a year resulted in serum 25OHD <90 ng/mL.^(^ ^29^ ^)^

There was no relationship between the administered amount of vitamin D and the level of urinary calcium excretion or hypercalcemia. Moreover, in half of these subjects, the hypercalciuria was transient. Adding further uncertainty to these data, however, was the observation that those receiving placebo experienced the same incidence of hypercalciuria. These data do not provide compelling support for the idea that such low‐dose regimens may be harmful. In fact, most experts agree that doses up to 4000 IU are probably safe.^(^ ^41^, ^95^ ^)^ A more compelling discussion focuses upon fall risk associated with high doses of vitamin D.^(^ ^69^, ^96^, ^97^ ^)^ Intermittent high boluses or administration of vitamin D to older individuals on a regular basis, associated with levels of 25OHD >45 ng/mL (>113 nmol/L), may lead to an increase risk of falls.^(^ ^79^ ^)^ Further research is needed to further clarify whether such 25OHD levels do indeed increase falls risk.

Skeletal health has also been a focus of recent studies related to adverse effects of high vitamin D dosing. In the Calgary study performed on healthy volunteers without osteoporosis whose mean baseline 25OHD was approximately 31 to 32 ng/mL, treatment with vitamin D for 3 years at a dose of 4000 IU/day or 10,000 IU/day, compared with 400 IU/day, resulted in statistically significant reduction in radial volumetric BMD.^(^ ^69^ ^)^ However, no significant differences in bone strength at either the radius or tibia were observed. Burt and colleagues concluded from this study that there was no benefit from doses of vitamin D at 4000 IU or higher as an adjunct to bone health.

### Vitamin D deficiency in acute illness

In the setting of acute illness, levels of 25OHD may be low because of the acute reduction in circulating DBP.^(^ ^98^ ^)^ Dilutional effects of acute fluid shifts in the intravascular space may also be a factor. Additionally, pre‐existing vitamin D nutritional status is also a factor. This latter point leads to the suggestion that correction of poor vitamin D status may decrease morbidity and mortality. Christopher and colleagues suggest that very high doses of vitamin D may be needed to see a benefit in patients in the intensive care unit.^(^ ^99^ ^)^ The higher doses may be needed because acutely ill patients may have secreted stress amounts of cortisol, which in turn could impair hepatic and renal hydroxylation of vitamin D.^(^ ^100^, ^101^ ^)^

### Vitamin D requirements during reproduction

There is a lack of consensus on the use of vitamin D during reproduction. On the one hand, maternal vitamin D requirements are not increased during pregnancy or lactation. The achieved 25OHD level is not affected by either reproductive state, and there is no evidence that women should maintain higher 25OHD levels when pregnant or breastfeeding as compared with the healthy nonpregnant ideal. On the other hand, poor maternal vitamin D status during pregnancy can affect fetal and neonatal health; so it certainly makes sense to ensure that maternal vitamin D status is optimized during pregnancy. This does not mean that women require “more” vitamin D when pregnant than when nonpregnant. During lactation maternal vitamin D status does not matter directly because little vitamin D gets into milk, and especially because RCTS have shown that across a range of low to high 25OHD levels, the calcium content of milk is independent of maternal vitamin D status. Breastfed babies need supplemental vitamin D, whereas formula‐fed babies get their vitamin D in the supplemented formula.

Although variability exists among different studies, evidence from RCTs and systematic reviews suggests a benefit of vitamin D repletion with up to 2000 IU/day for preeclampsia and gestational DM,^(^ ^102^ ^)^ as well as for neonatal outcomes.^(^ ^103^, ^104^, ^105^, ^106^, ^107^ ^)^ It seems reasonable to recommend that normal vitamin D status should be ascertained in pregnancy.

### The potential for a broad spectrum of cellular and organ activities under the influence of the vitamin D receptor

The vitamin D receptor (VDR) is present in virtually all cells and tissues. The 1a‐hydroxylase, CYP27B1, is also found throughout the body and in many cell types.^(^ ^108^ ^)^ It has been estimated that 3% to 10% of all genes in vertebrates, from zebrafish to mice to humans, are under the direct or indirect control of 1,25(OH)_2_D_3_.^(^ ^109^ ^)^ This evolutionary omnipresence suggests a fundamental role for vitamin D in the functioning of all organs. Experiments to delete this gene in a tissue‐specific manner in mice have confirmed this expectation. A sampling of tissue‐specific KO experiments shows that mammary glands are more prone to breast cancer,^(^ ^110^ ^)^ cardiac muscle develops cardiac hypertrophy,^(^ ^111^ ^)^ the liver becomes fatty (nonalcoholic fatty liver syndrome),^(^ ^112^, ^113^ ^)^ the prostate develops hyperplasia,^(^ ^114^ ^)^ atherosclerosis is accelerated,^(^ ^115^ ^)^ and mice become resistant to diet‐induced obesity.^(^ ^116^ ^)^ Conversely, overexpression of the VDR leads to obesity^(^ ^117^ ^)^ in the mouse, but not in humans.^(^ ^118^ ^)^ More work is needed to understand how these KO and overexpression models in mice relate to human pathophysiology.

Recent mortality data show an association between low 25OHD and increased risk of all‐cause mortality.^(^ ^119^, ^120^ ^)^ These findings were also observed in a European consortium.^(^ ^121^ ^)^ Several association analyses of overall mortality and cardiovascular mortality have shown a U‐shaped curve with increases at both ends.^(^ ^122^ ^)^ A meta‐analysis based on 75,000 patients from 38 supplementation trials also showed a small, but significant reduction in mortality (relative risk [RR], 0.94; 95% CI, 0.91–0.98).^(^ ^123^ ^)^

From Mendelian randomization studies examining the effects of vitamin D on autoimmune diseases, three independent findings show that decreased vitamin D levels (5% to 7% lower than normal levels) significantly increased the susceptibility to developing multiple sclerosis.^(^ ^124^, ^125^, ^126^ ^)^ Finally, one Mendelian randomization study showed an association with type 1 diabetes mellitus (T1DM) risk.^(^ ^127^ ^)^

The data on cancer in mice are also of interest. 1,25(OH)_2_D_3_‐deficient mice have a greater chance of developing cancer with increasing age,^(^ ^128^ ^)^ and an increased rate of proliferation in intestinal and breast cells. Although VDR‐null mice usually do not spontaneously develop more cancers, they are more likely to develop a range of malignancies, such as breast,^(^ ^129^ ^)^ colon,^(^ ^130^ ^)^ and skin^(^ ^131^, ^132^ ^)^ cancer, when exposed to oncogenes, loss of antioncogenes, or exposure to carcinogens or UVB light.^(^ ^133^, ^134^ ^)^ This is in line with the “cancer hypothesis,” where the risk of cancer development is associated with multiple events. Although these mice data appear to be compelling, Mendelian randomized studies in humans have not been supportive.^(^ ^135^ ^)^

### Potential links between vitamin D and major human diseases

Many cross‐sectional, observational, and retrospective studies have associated low vitamin D status with many human diseases.^(^ ^136^, ^137^, ^138^, ^139^ ^)^ In the aggregate, these reports suggest a pervasive influence of vitamin D on the health of most human organ systems. Preclinical evidence for a role of vitamin D in immune system regulation is perhaps strongest as the VDR and CYP27B1 are expressed in cells of both the innate and adaptive arms of the immune system. Moreover, CYP27B1 expression in immune cells is regulated by a complex innate immune and cytokine network.^(^ ^136^, ^137^, ^138^, ^139^, ^140^, ^141^ ^)^ There is widespread clinical evidence in both pediatric and adult populations that maintenance of vitamin D sufficiency should lower the incidence of infections of viral or bacterial origin.^(^ ^142^ ^)^ Accumulated evidence suggests that any role for vitamin D in autoimmune conditions would be preventive rather than therapeutic. One condition for which vitamin D supplementation may be of benefit is in the treatment of the inflammatory bowel condition Crohn disease, where meta‐analyses of a series of small‐scale trials suggest that supplementation reduces disease severity.^(^ ^143^, ^144^ ^)^ It would be important to conduct a large‐scale RCT in patients with Crohn disease to solidify these findings. Large‐scale RCTs are essential to determine whether the relationship between vitamin D deficiency and disease is causal or simply an association.

Another disorder to which vitamin D deficiency has been linked is DM. It has been shown that vitamin D prevents insulitis and the development of experimental DM by acting on the defective suppressor cellular function or by cytokine‐expression modulation. These observations have been confirmed, in part, by clinical findings showing that supplementation with vitamin D during early childhood may decrease the risk of developing T1DM.^(^ ^145^, ^146^ ^)^ However, further studies have not shown any significant effect of calcitriol supplementation on insulin secretion, insulin sensitivity, or insulin requirement or improvement in bone turnover in patients with newly diagnosed T1DM.^(^ ^147^, ^148^ ^)^

It is uncertain whether 25OHD levels in pregnancy or at birth reduce the risk of childhood T1DM. However, when the interaction with genetic variants is taken in consideration, higher 25OHD levels at birth predict a decreased risk of developing T1D or islet autoimmunity.^(^ ^149^, ^150^ ^)^ Both child or maternal VDR SNPs may lower VDR expression, and by consequence, inhibit T‐cell proliferation, thus increasing the risk of autoimmunity.

The recent Vitamin D Assessment (VIDA), Vitamin D and Omega‐3 (VITAL), and Vitamin D and Type 2 Diabetes (D2d) trials represent examples of attempts to translate these observations into clinical relevance.^(^ ^43^, ^151^, ^152^ ^)^

#### Cardiovascular disease

The VIDA trial tested the effect of a monthly dose of 100,000 IU of vitamin D3 compared with a placebo over a mean period of 3.4 years on cardiovascular disease among 5110 subjects.^(^ ^153^ ^)^ There was no statistical difference between the two groups. In the VITAL trial, there were no significant differences between the vitamin D and placebo groups in any individual cardiovascular event, such as myocardial ischemia, or in the composite cardiovascular end point.^(^ ^145^ ^)^

#### Cancer risk and survival

The much larger VITAL trial^(^ ^151^ ^)^ of 25,871 men and women aged over 50 years tested the effects of 2000 IU/day of vitamin D3 over 5 years on cardiovascular events and cancer. There was no significant difference between the vitamin D and placebo groups on the risk of developing any invasive cancer or individually in breast, prostate, or colorectal cancer. However, among those with a BMI <25, there was a significant reduction in any invasive cancer. Excluding the first 2 years of the study, there was also a reduction in the incidence of death from cancer. The study by Lappe and colleagues is noteworthy in this context; they show that calcium and vitamin D appeared to have an effect to reduce new cancer risk, but statistical significance was not achieved.^(^ ^154^ ^)^

A subsequent meta‐analysis by some of the invesitigators from the VITAL trial is noteworthy.^(^ ^155^ ^)^ Whereas VITAL appreciated a “signal” of improved survival in the vitamin‐D–supplemented group, the meta‐analysis of VITAL and several additional studies found a highly significant benefit on survival in the vitamin‐D–supplemented subjects, but again no benefit on risk of developing cancer. For total cancer incidence, 10 trials were included (6537 cases; 3 to 10 years of follow‐up; 54–135 nmol/L of attained levels of circulating 25OHD in the intervention group). The summary for cancer risk remained null across the subgroups tested, including when attained 25OHD levels exceeded 100 nmol/L. For total cancer mortality, five trials were included (1591 deaths; 3 to 10 years of follow‐up; 54 to 135 nmol/L of attained levels of circulating 25OHD in the intervention group). The summary RR was 0.87 (95% CI, 0.79–0.96; *p* = 0.005), which was largely attributable to interventions with daily dosing (as opposed to infrequent bolus dosing). Thus, this updated meta‐analysis of RCTs showed that vitamin D supplementation significantly reduced total cancer mortality, but did not reduce total cancer incidence. In the Torfadottir study, the goal was to explore whether prediagnostic circulating levels of 25OHD among older individuals were associated with overall and cancer‐specific survival after diagnosis.^(^ ^156^ ^)^ They used data from the AGES‐ (Gene/Environment Susceptibility‐) Reykjavik study on participants (*n* = 4619) without cancer at entry, when blood samples were taken for 25OHD standardized measurements. The association with cancer risk and all‐cause‐ and cancer‐specific mortality was assessed among those later diagnosed with cancer, comparing four 25OHD categories, using 50 to 69.9 nmol/L (20–28 ng/mL) as the reference category. Cancer was diagnosed in 919 participants on average 8.3 years after initial sampling. No association was observed between the reference group and other 25OHD groups and total cancer incidence. Mean age at diagnosis was 80.9 (± 5.7) years. Of those diagnosed, 552 died during follow‐up: 67% from cancer. Importantly, low prediagnostic levels of 25OHD <30 nmol/L (<12 ng/mL) were significantly associated with increased total mortality (hazard ratio [HR], 1.39; 95% CI, 1.03–1.88) and not significantly with cancer‐specific mortality (HR, 1.33; 95% CI, 0.93–1.90). Among patients surviving more than 2 years after diagnosis, higher prediagnostic 25OHD levels (≥70 nmol/L) were associated with lower risk of overall (HR, 0.68; 95% CI, 0.46–0.99) and cancer‐specific mortality (HR, 0.47; 95% CI, 0.26–0.99). It appeared that among elderly cancer patients, low prediagnostic serum 25OHD levels (<30 nmol/L [<12 ng/mL]) were associated with increased overall mortality.

#### Diabetes mellitus

The D2d trial examined the effect of vitamin D3 at 4000 IU/day on the development of overt DM among 2423 men and women aged >30 years, who had risk factors for DM. There was no difference in the probability of developing DM over this period between the vitamin D and placebo groups. However, a post hoc analysis in participants with a baseline 25OHD <12 ng/mL (or < 30 nmol/L) showed a 62% reduction in DM in the vitamin D group.

##### Pulmonary, blood pressure, and other effects

Additionally, from the VIDA trial, central blood pressure was significantly reduced in patients taking vitamin D supplementation (−7.5 mmHg, *p* = 0.03),^(^ ^157^ ^)^ and the number of patients taking NSAIDs was significantly reduced (RR, 0.87, *p* = 0.01).^(^ ^158^ ^)^ Furthermore, giving vitamin D to the normal population and to the vitamin D‐deficient population improves lung function,^(^ ^159^ ^)^ in line with a meta‐analysis,^(^ ^160^ ^)^ as well as reducing age‐related bone loss.^(^ ^161^, ^162^ ^)^ In an individual participant data meta‐analysis of 15 RCTs, daily or weekly supplementation in individuals with vitamin D deficiency, defined as a serum 25OHD level <10 ng/mL, reduced risk of acute respiratory infection by 30% (odds ratio, 0.30; 95% CI, 0.17–0.53).^(^ ^142^ ^)^

## Methodological Issues

**Fig 3:**
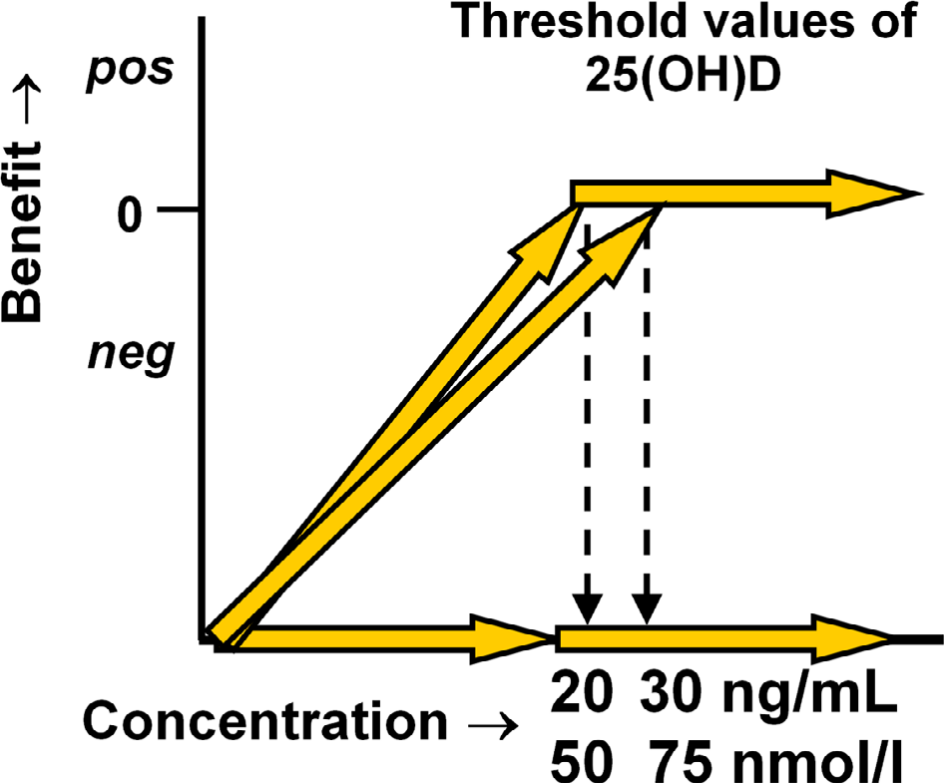
Threshold value of 25OHD where positive effects can be observed.

Unfortunately, what VIDA, VITAL, and D2d studies share is that baseline 25OHD levels were not deficient in the majority of participants. Mean baseline levels from VIDA (24.2 ng/mL or 60.5 nmol/L), VITAL (30.8 ng/mL or 77.0 nmol/L), and D2d (28.2 ng/mL or 70.5 nmol/L) were all within the normal range as defined by the Institute of Medicine. Levels below 20 ng/mL (50 nmol/L) were seen in only 33% of the VIDA, 12.7% of the VITAL, and 20.7% of the D2d populations. One important conclusion from these studies is that they did not show that a vitamin D‐deficient population would benefit by vitamin D repletion because the populations were already replete. As noted earlier, if subjects are already above the level for a threshold nutrient, giving more will not necessarily lead to beneficial effects. Therefore, it is not evidence‐based to claim, based on these studies, that vitamin D has no effects on cancer, the cardiovascular system, or the development of DM. A clue to the importance of this statement is the post hoc analysis of the D2d study in which subjects who were frankly vitamin D deficient, namely with levels of 25OHD <12 ng/mL (30 nmol/L) at baseline, were at reduced risk of developing DM (HR, 0.38; 95% CI, 0.18–0.80) if they were in the vitamin‐D–supplemented group (Fig. 3). Other clues are noted above with regard to blood pressure and pulmonary infections in which vitamin D did appear to have beneficial effects.

Another methodological issue is the duration of the studies. One has to consider how long prior to the development of cancer or cardiovascular disease or DM must an intervention have to influence the development of overt disease. For example, the Torfadottir study had a mean duration of 8.3 years from no sign of cancer to the cancer diagnosis.^(^ ^156^ ^)^ Is it likely that giving a vitamin D supplement for 5 years or less will alter the time course of cancer becoming apparent? Carcinogenesis is usually a slow process that proceeds undiagnosed in a stepwise fashion, perhaps for many years prior to the diagnosis. Thus, the finding that vitamin D supplementation can improve survival once the cancer is apparent, even if it does not reduce the risk of developing cancer over a 5‐year period of intervention, is nevertheless a major factor demonstrating the benefit of maintaining adequate levels of vitamin D.

Rigorous studies of vitamin D supplementation in subject cohorts deficient in vitamin D compared with adequate levels are needed to resolve the controversy surrounding potential/purported nonskeletal effects of vitamin D. For endpoints like cancer and cardiovascular disease, studies need to be carried out for longer duration than 5 years to clearly demonstrate the presence or absence of a benefit on risk. The benefit on cancer survival seems to be solidly demonstrated.

## Conclusions

In this review, we have highlighted areas of consensus and uncertainty with regard to vitamin D as a nutrient and regulator of cellular action. Although nutritional rickets is well‐defined and highly prevalent worldwide, a concerted global effort is required to eradicate this eminently curable condition. A better understanding of the endogenous production of vitamin D and the regulation of its metabolism along with the development of universally useful assays with proper quality control remain worthy goals. Although animal data provide a useful backdrop to hypotheses arguing for nonskeletal effects of vitamin D, human studies both in terms of several meta‐analyses, as well as recent RCTs, have not been designed to permit any definitive conclusions. We look forward to future well‐designed studies that can clearly establish the extent to which vitamin D's actions are pervasive and extend beyond the skeleton.

## Disclosure

The coauthors of this article have no conflicts to declare with regard to its content.

## Author contributions

**Andrea Giustina and John P. Bilezikian:** Conceptualization; data curation; methodology; supervision; writing‐original draft; writing‐review and editing. **Roger Bouillon:** Writing‐review and editing. **Neil Binkley:** Writing‐review and editing. **Christopher Sempos:** Writing‐review and editing. **Robert Adler:** Writing‐review and editing. **Jens Bollerslev:** Writing‐review and editing. **Bess Dawson‐Hughes:** Writing‐review and editing. **Peter Ebeling:** Writing‐review and editing. **David Feldman:** Writing‐review and editing. **Annemieke Heijboer:** Writing‐review and editing. **Glenville Jones:** Writing‐review and editing. **Christopher Kovacs:** Writing‐review and editing. **Marise Lazaretti‐Castro:** Writing‐review and editing. **Paul Lips:** Writing‐review and editing. **Claudio Marcocci:** Writing‐review and editing. **Salvatore Minisola:** Writing‐review and editing. **Nicola Napoli:** Writing‐review and editing. **René Rizzoli:** Writing‐review and editing. **Robert Scragg:** Writing‐review and editing. **John White:** Writing‐review and editing. **Anna Maria Formenti:** Writing‐review and editing.

### Peer review

The peer review history for this article is available at https://publons.com/publon/10.1002/jbm4.10417.
